# Mechanisms and Therapeutic Advances of PXR in Metabolic Diseases and Cancer

**DOI:** 10.3390/ijms26168029

**Published:** 2025-08-20

**Authors:** Yuanbo Bi, Sifan Liu, Lei Wang, Daiyin Peng, Weidong Chen, Yue Zhang, Yanyan Wang

**Affiliations:** School of Pharmacy, Anhui University of Chinese Medicine, Hefei 230012, China; biyuanbo05@163.com (Y.B.); 15391843023@163.com (S.L.); wanglei@ahtcm.edu.cn (L.W.); pengdaiyin@163.com (D.P.); wdchen@ahtcm.edu.cn (W.C.)

**Keywords:** pregnane X receptor (PXR), metabolic diseases, diabetes, obesity, metabolic dysfunction-associated steatotic liver disease (MASLD), cancer

## Abstract

The pregnane X receptor (PXR), a ligand-activated nuclear receptor, plays a central role in regulating the metabolism of both endogenous substances and xenobiotics. In recent years, increasing evidence has highlighted its involvement in chronic diseases, particularly metabolic disorders and cancer. PXR modulates drug-metabolizing enzymes, transporters, inflammatory factors, lipid metabolism, and immune-related pathways, contributing to the maintenance of hepatic–intestinal barrier homeostasis, energy metabolism, and inflammatory responses. Specifically, in type 2 diabetes mellitus (T2DM), PXR influences disease progression by regulating glucose metabolism and insulin sensitivity. In obesity, it affects adipogenesis and inflammatory processes. In atherosclerosis (AS), PXR exerts protective effects through cholesterol metabolism and anti-inflammatory actions. In metabolic dysfunction-associated steatotic liver disease (MASLD), it is closely associated with lipid synthesis, oxidative stress, and gut microbiota balance. Moreover, PXR plays dual roles in various cancers, including hepatocellular carcinoma, colorectal cancer, and breast cancer. Currently, PXR-targeted strategies, such as small molecule agonists and antagonists, represent promising therapeutic avenues for treating metabolic diseases and cancer. This review comprehensively summarizes the structural features, signaling pathways, and gene regulatory functions of PXR, as well as its role in metabolic diseases and cancer, providing insights into its therapeutic potential and future drug development challenges.

## 1. Introduction

**Figure 1:**
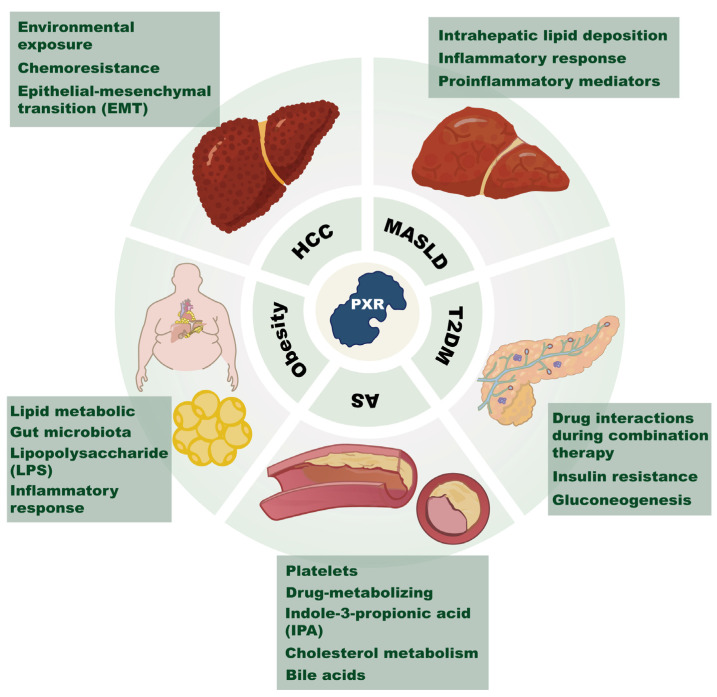
Schematic diagram of PXR’s role in metabolic diseases and cancer. This figure highlights PXR’s significant role across various chronic metabolic disorders. It shows PXR’s involvement in lipid and gut microbiota regulation in obesity, as well as its role in AS (atherosclerosis). For hepatic issues like HCC (Hepatocellular carcinoma) and MASLD (metabolic dysfunction-associated steatotic liver disease), PXR affects lipid accumulation and inflammation in the liver. In drug metabolism, PXR adjusts enzymes and transporters, impacting how drugs interact and insulin resistance. In terms of cardiovascular health, PXR influences glucose metabolism, key in T2DM (type 2 diabetes mellitus) and gluconeogenesis. This points to PXR’s value as a target for treating these widespread health issues.

The classical definition of nuclear receptors (NRs) refers to ligand-activated transcription factors. Advances in human genome sequencing and cryo-electron microscopy techniques have elucidated 48 members of the NR superfamily at both gene and protein levels [1]. According to their ligand types, NRs can be classified into steroid hormone receptors, non-steroid hormone receptors such as thyroid hormone receptors, and orphan nuclear receptors [2]. Pregnane X receptor (PXR) was initially identified as an orphan nuclear receptor; however, subsequent studies have demonstrated that it can be modulated by diverse endogenous and exogenous ligands [3,4]. Extensive evidence indicates that NRs play critical roles in metabolism and disease pathogenesis, including chronic metabolic disorders that threaten human health, such as type 2 diabetes mellitus (T2DM), obesity, atherosclerosis (AS), metabolic dysfunction-associated fatty liver disease (MASLD), and cancer [5] (Figure 1). Over recent decades, these chronic metabolic diseases have become a global health burden, with epidemiological data showing a sharp increase in disability-adjusted life years and mortality rates for several metabolic conditions [6].

As a member of the NR superfamily, PXR has attracted significant attention for its regulatory functions in chronic diseases. Numerous studies have demonstrated PXR’s therapeutic potential in T2DM, obesity, cardiovascular diseases, MASLD, and cancer [7]. Compared with other nuclear receptors, PXR possesses a unique protein structure characterized by a large and flexible ligand-binding pocket, enabling it to interact with a wide array of ligands [8]. Furthermore, PXR can form structurally distinctive complexes such as heterodimers or tetramers, which enhance its affinity for coactivators and modulate downstream signaling pathways [9]. Additionally, PXR regulates the expression of multiple drug-metabolizing enzymes and transport proteins and is a key factor contributing to insulin resistance and aberrant gluconeogenesis [10,11]. These distinctive features have guided in-depth investigations into PXR’s pivotal role in drug metabolism, glucose metabolism, lipid metabolism, and other metabolic disorders. The aim of this review is to summarize current understanding of PXR’s regulatory mechanisms in chronic diseases, evaluate its potential as a therapeutic target, and provide perspectives for future research directions on PXR.

## 2. Molecular Characteristics and Biological Functional Mechanisms of PXR

### 2.1. Structure and Function of PXR

PXR is a member of the nuclear receptor family, located on the human chromosome 3q12-q13.3 region, and encoded by 434 amino acids [12]. Members of the nuclear receptor family typically share conserved structural domains, and PXR is no exception. It consists of a DNA-binding domain (DBD), a ligand-binding domain (LBD), a hinge region, an N-terminal ligand-independent transactivation domain AF-1, and a ligand-dependent transactivation domain AF-2 located at the C-terminus within the LBD [13]. Among these, the structures of the DBD and LBD are especially critical for elucidating PXR’s mechanism of action. Within the DBD, distinct components have been identified, including two zinc finger (ZF) motifs and a bipartite nuclear localization signal. The zinc finger structures enable binding to response elements in downstream target gene promoters and distal enhancers. Furthermore, studies have demonstrated that these two zinc finger motifs exhibit an irreplaceable role in binding to mitotic chromatin, thereby fully exerting the functions of the DBD domain [14,15]. In addition, human PXR LBD (hPXR-LBD) adopts a three-layered α-helical sandwich architecture composed of α1/α3, α4/α5/α8/α9, and α7/α10 helices. Besides the α-helices, the structure contains five antiparallel β-strands, including β2, β3, β4, β1, and β1′ [16]. Distinct from other nuclear receptors, hPXR-LBD contains a unique insertion of approximately 50 residues (amino acids 177–228), which includes α2, β1, β1′, and a tryptophan zipper (Trp-Zip) motif [8,17]. The ligand-dependent transactivation domain AF-2 is located at the C-terminal end of hPXR-LBD, terminating in an α-helix (αAF). The interface of the αAF helix exhibits strong polarity and hydrophobicity, favoring the recruitment of transcriptional coactivators [18]. These α-helices and β-strands collectively form the ligand-binding pocket. The ligand-binding cavity of hPXR-LBD comprises 28 amino acid residues and presents as a predominantly hydrophobic pocket shaped by the interplay of hydrophobic and polar residues [16].

In addition to elucidating the structure of the ligand-free PXR, considerable attention has been given to the unique structures and functions of PXR complexes. Structural analyses have revealed various PXR-containing assemblies, including PXR homodimers, heterodimers formed between PXR and the 9-cis retinoic acid receptor (also known as retinoid X receptor, RXR), binary complexes of PXR with different ligands, ternary complexes comprising PXR, ligand, and transcriptional coactivators, as well as heterotetramers formed by the ligand-binding domains of PXR and RXRα [9,19,20]. The diverse interacting partners of PXR confer distinct mechanistic roles; for example, monomeric PXR alone is inefficient at recruiting coactivators, whereas PXR-associated complexes significantly enhance coactivator binding affinity [19]. These activated complexes can promote nuclear translocation and directly bind DNA to regulate gene transcription, such as the induction of cytochrome P450 3A (CYP3A) expression, thereby accelerating the metabolism of certain drugs [21]. This provides a theoretical foundation and broad prospects for investigating the mechanisms underlying PXR-mediated drug metabolism and drug–drug interactions.

Owing to the unique characteristics of the human PXR ligand-binding pocket, it can recognize and bind a wide variety of endogenous and exogenous compounds. For instance, hPXR can be activated by rifampicin and the cholesterol-lowering drug SR12813 [22]. Although this broad ligand-binding capability might suggest a non-specific receptor profile, interspecies differences in PXR agonist specificity are well documented. For example, mouse PXR is activated by pregnenolone 16α-carbonitrile (PCN) but is not responsive to rifampicin or SR12813 [23]. Studies indicate that a limited number of polar residues within the ligand-binding cavity are key determinants of these interspecies differences. By rationally modifying the ligand-binding pocket via targeted mutations, mouse PXR mutants have been engineered to respond to hPXR agonists [24]. We now note that even when mouse PXR is engineered to respond to human ligands, transcriptional responses (magnitude, kinetics) may still differ due to species-divergent promoter architecture and coactivator recruitment. We also point out that humanized PXR mice may only recapitulate some of the human responses that are relevant to the research focus, posing challenges for translational studies.

### 2.2. PXR Signaling Pathways

As described above, PXR can form homodimers and heterodimers or heterotetramers with RXR, which are closely related to the unique amino acid residues and structural motifs within the PXR LBD. The PXR/PXR homodimer interface is formed by antiparallel interactions between the β1′ strands located on the insertion segments of each PXR monomer. Tryptophan and tyrosine residues at the interface interlock in a characteristic “tryptophan zipper” (Trp-Zip) motif, resulting in a 10-stranded antiparallel intermolecular β-sheet [19]. This dimerization stabilizes the β1–β1′ region, thereby tightening the connection between the β-sheet and the α10 helix as well as the pseudohalix α2 within the activation function (AF) domain of PXR. Additionally, studies indicate that residues at the α12 interface within the AF domain critically determine PXR’s response to ligands; mutations at α12 often lead to altered agonist or antagonist effects compared to the wild-type receptor [25]. Thus, it can be inferred that mutations in key residues at the dimer interface or within the AF-2 domain severely impair responsiveness to agonists and antagonists by disrupting the interaction between PXR-LBD and transcriptional coactivators. This finding offers novel insights for modulating PXR-mediated metabolic activity during pharmacotherapy.

The formation of the PXR/RXR heterodimer primarily involves specific residues of hPXR and human RXRα (hRXRα), which interact via electrostatic forces, polar contacts, and hydrophobic interactions. Because the surfaces mediating PXR homodimerization and PXR-RXRα heterodimerization do not overlap, a structurally feasible PXR-RXRα heterotetramer model can be generated theoretically. Previous research demonstrates that within the PXR/RXRα heterotetramer, both PXR and RXR ligand-binding domains exhibit markedly enhanced affinity for the coactivator SRC-1 [9]. This suggests that the transcriptional regulatory capacity of PXR is likely associated with the enhanced recruitment of coactivators by the PXR/RXRα complex. This phenomenon can be explained by the fact that PXR/RXR forms a permissive heterodimer, meaning that the heterodimer can be activated by ligands binding to RXR or its partner receptor. When ligands for both PXR and RXR are present, a synergistic response stronger than that induced by either ligand alone occurs [26]. Notably, SRC-1 serves as a common coactivator for both PXR and RXR.

### 2.3. PXR Target Genes

Members of the cytochrome P450 (CYP) family play critical roles in the oxidative metabolism of various endogenous substances and xenobiotics such as drugs and environmental pollutants. Among them, CYP3A4 is especially important in human drug metabolism, catalyzing the metabolism of over 50% of clinically used drugs [27]. As a nuclear receptor, PXR primarily exerts its regulatory function by forming a complex upon ligand activation, which translocates into the nucleus and directly binds downstream target gene DNA via its DNA-binding domain (DBD) to regulate gene expression [28]. Previous studies have shown that the heterodimer formed by human PXR and RXR can specifically bind to the ER6-type PXR response element (PXRE) in the CYP3A4 promoter, competing effectively with oligonucleotides to interact with DNA [21]. Besides CYP3A, the expression of CYP2B6 in the liver varies significantly among individuals and has also attracted research interest. It was found that PXR/RXRα and CAR/RXRα heterodimers bind synergistically to distal modules XREM (NR3 and NR8 motifs) and proximal module PBREM (NR1 and NR2 motifs) in the CYP2B6 promoter to jointly mediate transcriptional activation of the CYP2B6 gene [29]. These findings collectively indicate that PXR plays a vital role in regulating cytochrome P450 family gene expression, thus providing a mechanistic basis for drug metabolism research and new drug development.

In addition to regulating phase I metabolic enzymes, PXR also modulates key transport proteins that play important roles in human physiology. One such transporter is P-glycoprotein (P-gp), encoded by the MDR1 gene, which is crucial for the absorption and metabolism of many xenobiotics [30]. Research has identified a distal module approximately 28 kb upstream of the MDR1 promoter containing three DR4 motifs (DR4(I), DR4(II), DR4(III)), one DR3 motif, and an ER6 motif, which form a cluster of potential PXR binding sites. The PXR/RXRα heterodimer regulates MDR1 expression by binding specifically to the DR4(I) motif, thereby enhancing intestinal barrier function and preventing absorption of harmful substances [31]. Another important transporter regulated by PXR is the multidrug resistance-associated protein 2 (MRP2, also known as ABCC2). MRP2 is a member of the ATP-binding cassette (ABC) transporter family, primarily localized to the canalicular (apical) membrane of hepatocytes, and is involved in the transport of organic anions and xenobiotics [32]. Mutations in the MRP2 gene impair the secretion of conjugates of xenobiotics and endogenous compounds such as bilirubin into bile. Research by Heidi R. Kast et al. demonstrated that three heterodimers—PXR/RXR, FXR/RXR, and CAR/RXR—can bind to a newly identified ER-8 response element in the proximal promoter of the MRP2 gene, thereby activating its expression [33].

## 3. PXR in Metabolic Diseases and Cancer

### 3.1. T2DM

**Figure 2:**
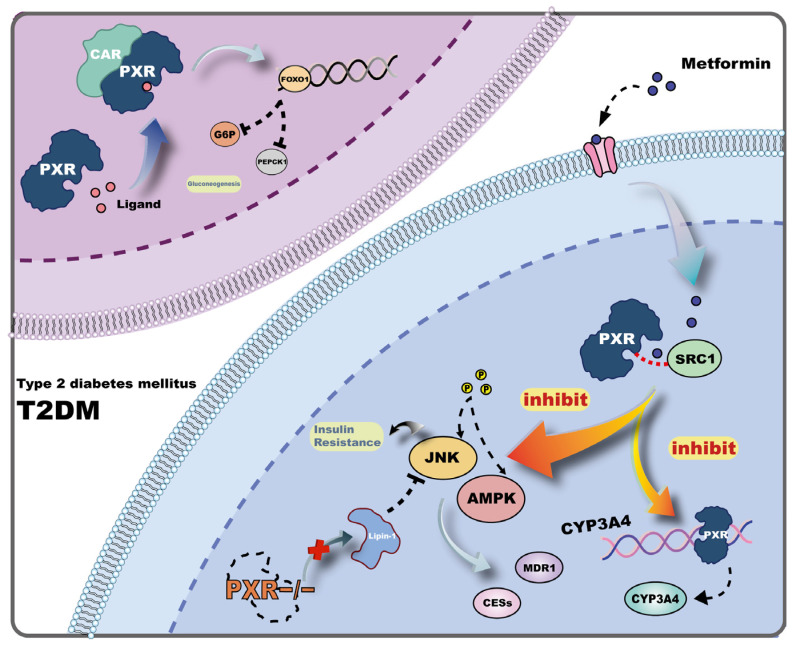
Mechanisms of PXR’s action in T2DM. This figure illustrates the complex interplay of PXR in the context of T2DM and drug metabolism. In T2DM, PXR activation can exacerbate insulin resistance through the JNK and AMPK signaling pathways, leading to reduced expression of PXR target genes such as MDR1 (multidrug resistance protein 1). The figure also shows that metformin, a commonly used drug for T2DM, can inhibit PXR activity, thereby affecting the expression of CYP3A4 and potentially altering the metabolism of co-administered drugs. The inhibition of PXR by metformin disrupts the interaction between PXR and SRC1 (steroid receptor coactivator-1), which is crucial for PXR’s role in drug metabolism. Furthermore, PXR’s activation is associated with the downregulation of key gluconeogenic enzymes such as G6P (glucose-6-phosphatase) and PEPCK1 (phosphoenolpyruvate carboxykinase 1), as well as the reduction of GLUT2 (glucose transporter 2) expression, which impairs insulin secretion and hepatic glucose metabolism. These mechanisms contribute to the dysregulation of glucose homeostasis and the development of diabetes.

Diabetes is a metabolic disorder characterized by insulin resistance and/or defects in insulin secretion. Based on specific pathological mechanisms, it is generally classified into two major types: type 1 diabetes, caused by autoimmune destruction of pancreatic β-cells resulting in insufficient insulin secretion, and type 2 diabetes, where hyperglycemia induces both insulin resistance in peripheral tissues and impaired insulin secretion [34]. T2DM is pathologically complex and often requires combination pharmacotherapy. Metformin remains a widely used first-line drug due to its potent glucose-lowering effects. However, the use of multiple drugs raises concerns regarding potential drug–drug interactions, and PXR plays a key role in the metabolism of xenobiotics. By regulating the expression of drug-metabolizing enzymes and proteins such as multidrug resistance protein 1 (MDR1), PXR significantly influences drug metabolism and clearance [35,36]. Studies have demonstrated that in human hepatocytes, metformin can inhibit the expression of CYP3A4 by disrupting the interaction between PXR and the steroid receptor coactivator-1 (SRC1), thereby altering the metabolism of co-administered drugs [37]. In another study conducted at the mice level, metformin suppressed the expression of hepatic carboxylesterases (CESs) by co-modulating PXR activity along with the AMPK and JNK signaling pathways, leading to reduced expression of the PXR target gene MDR1 [38]. These findings suggest that PXR plays an important role in drug interactions during combination therapy for T2DM and provide a theoretical basis for understanding the mechanisms underlying metformin-mediated pharmacokinetic effects. Beyond these mechanisms, crosstalk between PXR and the JNK signaling pathway is central to the development of insulin resistance. Overexpression of the hepatic PXR target gene Lipin-1, along with cellular stress, promotes phosphorylation of c-Jun N-terminal kinase (JNK), whereas PXR-knockout (PXR-KO) mice exhibit suppressed JNK phosphorylation and show significant alleviation of high-fat diet (HFD)-induced insulin resistance [10] (Figure 2). Persistent insulin resistance leads to substantial metabolic disruptions, such as increased hepatic gluconeogenesis and glycogenolysis, further exacerbating hyperglycemia [39,40]. Notably, aberrant activation of PXR has been closely linked to enhanced hepatic glucose metabolism. This relationship may be due to PXR-induced activation of multiple synergistic pathways involved in gluconeogenic regulation. Previous studies have demonstrated that ligand-dependent activation of PXR can crosstalk with constitutive androstane receptor (CAR), and both form heterodimers with RXR, cooperatively repressing the expression of forkhead box protein O1 (FOXO1) [41]. This suppression leads to downregulation of key gluconeogenic enzymes, glucose-6-phosphatase (G6P) and phosphoenolpyruvate carboxykinase 1 (PEPCK1), disrupting normal gluconeogenesis [11,42]. Furthermore, in vitro studies using mice hepatocytes have revealed that PXR activation reduces the expression of peroxisome proliferator-activated receptor gamma coactivator 1-alpha (PGC-1α), thereby exacerbating insulin resistance [43]. In addition, PXR activation downregulates hepatocyte nuclear factor 4 alpha (HNF4α), a transcriptional activator of glucose transporter 2 (GLUT2) in hepatocytes. Consequently, GLUT2 expression is reduced, leading to impaired insulin secretion and hepatic glucose metabolism [44,45] (Figure 2). Through these complex mechanisms, glucose homeostasis becomes severely dysregulated, resulting in sharply elevated blood glucose levels and the onset of diabetes.

With growing research attention, the understanding of non-genetic contributors to diabetes has expanded. Environmental and lifestyle factors have been increasingly recognized as major contributors. Various persistent environmental pollutants have been linked to increased diabetes incidence. Among them, polychlorinated biphenyls (PCBs) are classic examples. Studies have shown that non-dioxin-like PCBs (NDL-PCBs) may act as ligands for PXR, thereby activating it and promoting hepatic gluconeogenesis, ultimately leading to insulin resistance [46]. Another pollutant, polybrominated diphenyl ethers (PBDEs), may affect the levels of the gut microbial metabolite indole-3-propionic acid (IPA), which in turn modulates PXR activation and contributes to diabetes development [47]. These findings highlight environmental pollutants as important pathogenic factors in PXR-mediated diabetes, underscoring the significance of minimizing exposure to such agents and adopting healthier lifestyles to mitigate disease risk.

### 3.2. Obesity

Obesity is generally defined as excessive fat accumulation that impairs health. It is widely recognized to result from an imbalance between energy intake and expenditure, with HFD being a major contributing factor [48]. In addition, genetic predisposition, unfavorable environmental conditions, and unhealthy lifestyles also play critical roles in driving the pathogenesis of obesity. Dysfunction of adipose tissue leads to systemic metabolic disorders, particularly affecting lipid metabolism. Obesity-associated dysregulation of lipolysis, ectopic fat deposition, and chronic inflammation collectively promote the development of insulin resistance, hypertension, and other cardiovascular diseases [49].

**Figure 3:**
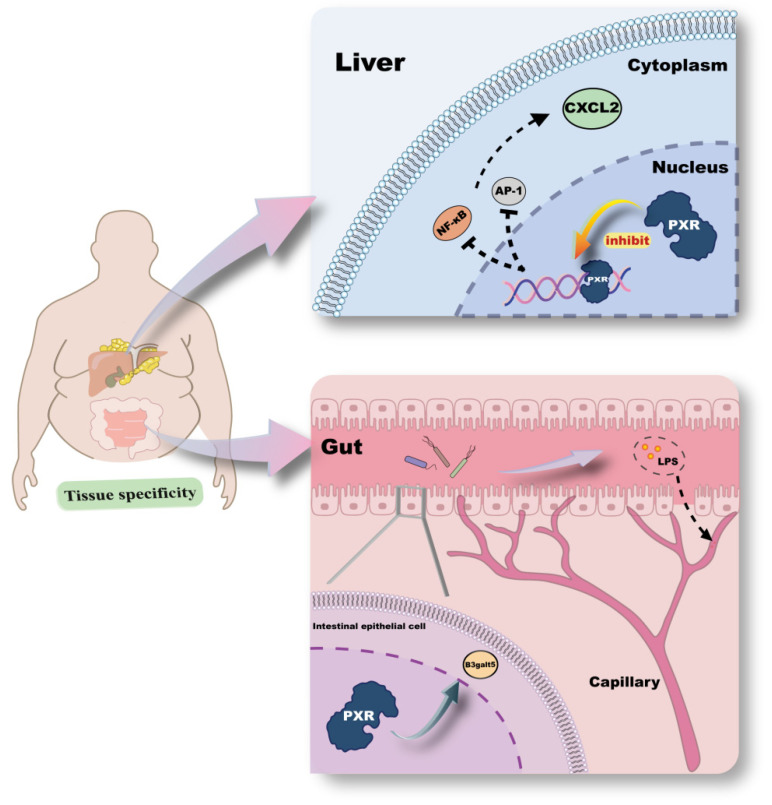
Mechanisms of PXR’s action in obesity. This figure highlights the tissue-specific functions of PXR in the liver and gut, illustrating its role in maintaining metabolic balance. In the liver, PXR activation inhibits the transcription factors NF-κB (nuclear factor-kappa B) and AP-1 (activator protein-1), leading to the downregulation of the inflammatory chemokine CXCL2. Conversely, in the gut, PXR activation upregulates the expression of its target gene B3galt5 (β-1,3-galactosyltransferase 5), which is crucial for maintaining the integrity of the intestinal barrier. The figure shows PXR’s protective role against increased intestinal permeability, which could otherwise allow harmful substances like LPS (lipopolysaccharide) to enter the bloodstream, triggering systemic inflammation and exacerbating obesity. These tissue-specific regulatory roles of PXR underscore its potential as a target for modulating metabolic disorders linked to obesity, offering insights into developing personalized therapeutic strategies.

The intestine is a key organ for lipid absorption and plays an essential role in maintaining lipid metabolic homeostasis [50]. Convergent evidence from human biopsy samples and mice model studies demonstrates that activation of intestinal PXR upregulates the expression of its target gene, β-1,3-galactosyltransferase 5 (B3galt5), thereby preserving the integrity of the intestinal barrier and preventing metabolic disorders induced by barrier dysfunction [51]. It can be inferred that suppression of PXR-mediated regulation of B3galt5 may increase intestinal permeability. Under chronic HFD conditions, this may lead to gut microbiota dysbiosis, allowing harmful substances such as lipopolysaccharide (LPS) to translocate into the bloodstream and trigger systemic inflammation, further aggravating the pathological state of obesity [52] (Figure 3). Encouragingly, activation of hepatic PXR in mice has been reported to inhibit the transcription of nuclear factor-kappa B (NF-κB) and activator protein-1 (AP-1), thereby downregulating the expression of the inflammatory chemokine CXCL2 and effectively mitigating the inflammatory response [53] (Figure 3). These findings highlight the importance of PXR’s tissue-specific regulatory roles, which may synergistically modulate metabolic disorders associated with obesity. Beyond tissue specificity within the same individual, the mechanisms of obesity may also vary among individuals due to factors such as sex and genetic background. A 16-week HFD intervention study revealed that PXR-mediated obesity and associated hepatic pathology occurred only in male mice, with no significant changes observed in female mice [54]. However, in another 52-week long-term HFD study, female mice also exhibited marked pathological changes, while PXR-knockout (PXR-KO) female mice were protected against HFD-induced obesity [55]. These results suggest that PXR also mediates obesity in females, although its underlying mechanisms may differ due to sex-specific effects. Such findings deepen our understanding of the complex regulatory functions of PXR and offer new perspectives for developing individualized therapeutic strategies. Currently, several therapeutic options are available for the treatment of obesity, including pharmacological interventions, endoscopic procedures, and bariatric surgery [56], all of which can effectively alleviate metabolic abnormalities. Based on the PXR-mediated mechanisms in obesity, targeting PXR represents a promising direction for new drug development. However, given the bidirectional regulatory effects of PXR, it is crucial to carefully evaluate the distinct outcomes of its activation and antagonism. As the saying goes, “Every coin has two sides.”

### 3.3. Atherosclerosis

The primary cause of atherosclerosis is hypercholesterolemia [57]. An unhealthy dietary pattern, characterized by excessive fat intake, significantly elevates plasma low-density lipoprotein cholesterol (LDL-C) levels, leading to diffuse intimal thickening (DIT) and the appearance of fatty streaks—hallmark features of early vascular lesions [58,59]. Lipid deposition, along with other biological processes such as macrophage uptake of oxidized LDL (Ox-LDL) and transformation into foam cells, contributes to the formation of advanced atherosclerotic plaques. The instability of these plaques can result in rupture, which in turn activates the coagulation system and promotes thrombus formation [60,61].

**Figure 4:**
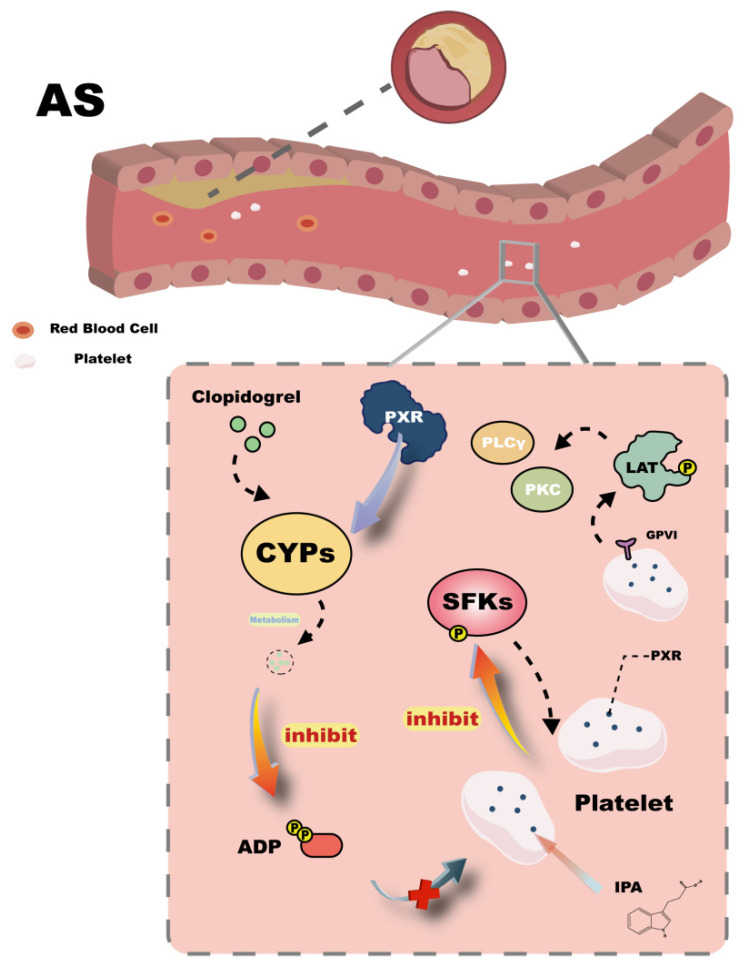
Mechanisms of PXR’s action in Atherosclerosis. This figure highlights how PXR agonists, such as SR12813, can inhibit SFKs (Src family kinases) phosphorylation, thereby reducing platelet activation. The figure also indicates that PXR influences the expression of drug-metabolizing enzymes like CYPs (cytochrome P450), which are necessary for the metabolic activation of antiplatelet agents like clopidogrel. This activation leads to the inhibition of ADP-induced platelet aggregation, as depicted by the inhibitory arrows pointing towards ADP and SFKs. Additionally, the figure suggests that dietary interventions, such as IF (intermittent fasting), can increase the production of IPA (indole-3-propionic acid), a PXR ligand. This figure underscores the significance of PXR in both direct and indirect regulation of platelet activity, offering insights into potential therapeutic strategies for preventing atherosclerosis.

In the bloodstream, platelets are critical targets in the regulation of cardiovascular diseases. They play an essential role in thrombus formation and atherosclerosis progression and are capable of expressing various nuclear receptors—an observation that has prompted research into their possible interactions [62,63]. In platelets, inhibition of phosphorylation of Src family kinases (SFKs) reduces platelet activation. Studies have shown that the PXR agonist SR12813 can suppress Src phosphorylation, and several PXR ligands are also capable of inhibiting platelet activation [64] (Figure 4). These findings support the hypothesis that PXR may regulate platelet activation directly or indirectly and provide a basis for further investigation. Moreover, PXR has been shown to modulate the expression of drug-metabolizing enzymes such as CYP3A, CYP2B6, CYP2C9, and CYP2C19, thereby indirectly influencing platelet activation. For instance, clopidogrel, an antiplatelet agent, requires metabolic activation by these enzymes to produce an active metabolite that inhibits ADP-induced platelet activation [65] (Figure 4). This action is attributed to PXR expression in vascular tissue, highlighting that, in addition to the liver, blood vessels serve as significant sites for drug metabolism. Consequently, potential drug–drug interactions within the vasculature may act as unrecognized contributors to cardiovascular disease risk and should be considered in drug development. Given the inherent risks associated with pharmacotherapy, prevention strategies are crucial. Healthy dietary habits can favorably influence platelet activity and reduce atherosclerosis risk. Intermittent fasting (IF), for example, is a dietary regimen that modulates the gut microbiota to increase the production of IPA, a well-known PXR ligand. Elevated IPA levels in plasma can bind platelet PXR, inhibiting downstream SFKs phosphorylation and the GPVI signaling cascade (LAT/PLCγ/PKC/Ca^2+^), thereby suppressing platelet activation [66] (Figure 4). These findings underscore the positive role of PXR-mediated inhibition of platelet activation in preventing atherosclerosis onset.

Beyond preventing life-threatening thrombotic complications in advanced stages of atherosclerosis, maintaining normal plasma cholesterol levels is key to halting early-stage vascular lipid deposition and preventing disease progression. The intestine plays a vital role in cholesterol metabolism [67]. In mouse studies, activation of PXR with the ligand pregnenolone 16α-carbonitrile (PCN) elevated total and LDL cholesterol levels [68]. Another study based on in vitro HepG2 cells found that this effect is closely associated with the upregulation of lipogenic genes. Cannabidiol (CBD), a selective PXR agonist, induced PXR activation and significantly increased the expression of NPC1L1, MTP, and CD36—three key genes involved in cholesterol uptake—thus promoting intestinal cholesterol absorption and contributing to hypercholesterolemia and macrophage foam cell formation in atherosclerosis [69,70]. Therefore, controlling dyslipidemia is essential. Ligand-dependent activation of PXR reduces its binding to heat shock protein 90 (HSP90), while increasing the binding of sterol regulatory element-binding protein 2 (SREBP2) to HSP90, forming a stable complex that facilitates nuclear translocation of both PXR and SREBP2. This in turn upregulates downstream NPC1L1 and HMGCR expression, promoting cholesterol synthesis [28,71]. Additionally, PXR activation may affect the structural stability of INSIG1 protein, thereby activating SREBP2 and increasing expression of cholesterol synthesis enzymes such as PCSK9, HMGCR, and SQLE. Overexpression of PCSK9 reduces LDL receptor levels on hepatocyte membranes and impairs cholesterol clearance [72,73]. Based on these mechanisms, it is reasonable to hypothesize that inhibiting PXR activation may reduce cholesterol synthesis, lower intracellular cholesterol levels, and enhance plasma cholesterol clearance, thereby maintaining cholesterol homeostasis. Supporting this hypothesis, PXR antagonists such as ketoconazole and chlorogenic acid have been shown to suppress PXR activation and synergistically enhance lipid-lowering effects [28].

Excess cholesterol can also be converted into bile acids and excreted via the intestine, which is a major pathway for eliminating excess cholesterol. PXR often works in concert with other nuclear receptors to regulate bile acid synthesis. A novel ligand, MI-883, designed to antagonize PXR while activating CAR, was found in animal studies to increase the expression of bile acid-synthesizing enzymes (e.g., Cyp7a1, Cyp8b1) and reduce the expression of ileal transporters (Asbt, Ostα), thereby suppressing bile acid reabsorption and significantly increasing fecal bile acid excretion [74]. Another study reported that PXR inhibition could secondarily enhance FXR activity, thereby modulating downstream LXRα and suppressing bile acid synthesis [75]. Conversely, PXR activation may stimulate bile acid synthesis via the FXR–LXRα axis, promote bile acid excretion, and ultimately lower plasma cholesterol levels, reducing the risk of atherosclerosis.

### 3.4. MASLD

**Figure 5:**
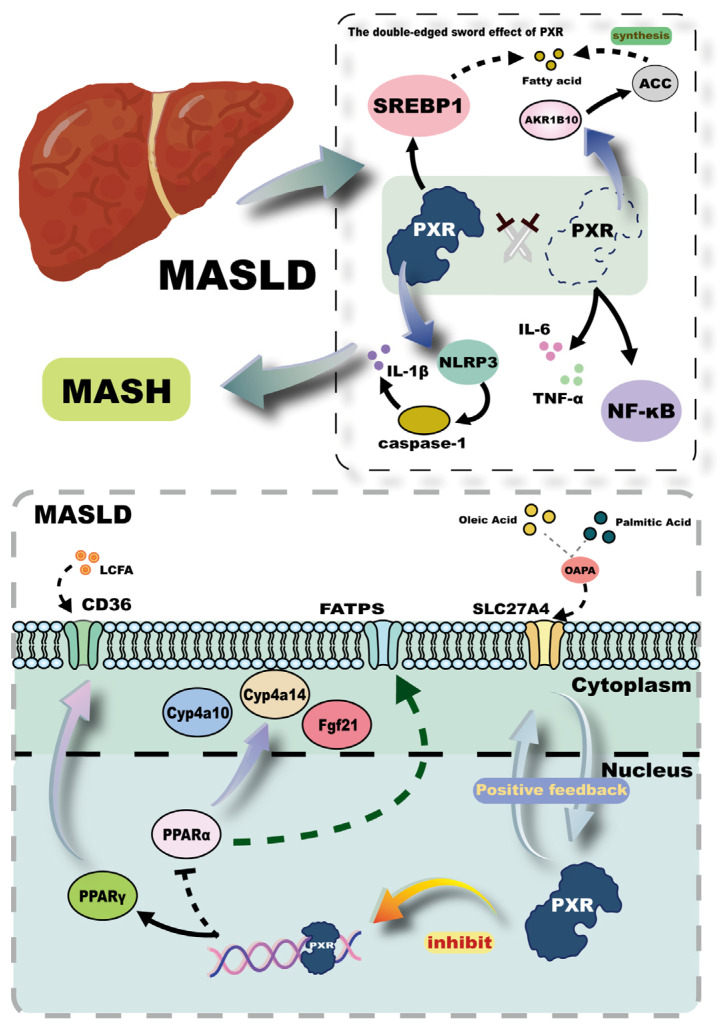
Mechanisms of PXR’s action in MASLD. This figure depicts PXR’s complex involvement in the pathogenesis of MASLD, highlighting its dual role in hepatic lipid metabolism and inflammation. In the liver, PXR activation forms a positive feedback loop with SLC27A4 (solute carrier family 27 member 4), enhancing fatty acid uptake and contributing to lipid accumulation and inflammation. Additionally, PXR influences the expression of PPARα (peroxisome proliferator-activated receptor alpha) and PPARγ (peroxisome proliferator-activated receptor gamma), key regulators of fatty acid oxidation and uptake, respectively. The figure also illustrates the paradoxical effects of PXR on inflammation, where both its inhibition and activation can promote inflammatory responses through different pathways, such as the NLRP3 inflammasome and the regulation of proinflammatory mediators like IL-6 (interleukin-6), TNF-α (tumor necrosis factor-α), and NF-κB (nuclear factor-kappa B). Furthermore, PXR’s activation in hepatocytes can upregulate SREBP1 (sterol regulatory element-binding protein 1), promoting lipogenesis, while PXR knockdown can increase AKR1B10 (aldo-keto reductase 1B10), enhancing fatty acid synthesis and contributing to steatosis.

MASLD, previously known as non-alcoholic fatty liver disease (NAFLD), reflects a nomenclature shift based on updated pathological insights. It is now well-recognized that hepatic lipid accumulation resulting from metabolic dysregulation is a primary driver of disease progression [76]. Elucidating the underlying mechanisms of metabolic dysfunction and identifying potential therapeutic targets are crucial for improving MASLD outcomes. One important strategy involves the reduction of intrahepatic lipid deposition, a process in which the PXR has been implicated. In primary human hepatocytes (MPHs) derived from MASLD patients, oleic acid and palmitic acid (OAPA) exposure has been shown to upregulate the expression of solute carrier family 27 member 4 (SLC27A4), a key membrane protein that facilitates the uptake and activation of fatty acids. This upregulation promotes intracellular lipid accumulation and inflammation. Notably, increased SLC27A4 expression enhances PXR activity, and PXR activation, in turn, further induces SLC27A4 expression, forming a positive feedback loop [77,78] (Figure 5). Therefore, silencing or pharmacological inhibition of PXR significantly reduces the pro-steatotic effects of SLC27A4 in MASLD. Additionally, the crosstalk between PXR and peroxisome proliferator-activated receptors (PPARs), both members of the nuclear receptor superfamily, plays a central role in hepatic lipid homeostasis. PPARα, a subtype of PPARs, regulates fatty acid oxidation by modulating the expression of membrane-associated fatty acid transport proteins (FATPs) and key enzymes in peroxisomal β-oxidation [79,80]. In a study employing both wild-type mice and PXR-knockout (PXR-KO) mice, it has been demonstrated that the activation of hepatic PXR suppresses the hepatic expression of PPARα and its downstream targets, such as Fgf21, Cyp4a10, and Cyp4a14, thereby exacerbating hepatic steatosis [81,82]. Furthermore, PPARγ, another PPAR subtype, directly regulates the expression of CD36, a fatty acid translocase that enhances the uptake of long-chain fatty acids (LCFAs). PXR activation has been shown to directly bind to the promoter region of the PPARγ gene, upregulating its expression and subsequently increasing CD36 expression, thereby augmenting hepatic LCFAs uptake (Figure 5). This regulatory mechanism exhibits liver specificity and contributes to hepatic lipid accumulation [83,84].

Excessive accumulation of toxic lipids in the liver impairs hepatic homeostasis, triggering immune responses that lead to local inflammation and eventually progress to metabolic dysfunction-associated steatohepatitis (MASH). Several studies conducted at the mice level and in vitro human hepatocytes have indicated that inhibition of PXR leads to the upregulation of proinflammatory mediators such as interleukin-6 (IL-6), tumor necrosis factor-α (TNF-α), and NF-κB, thereby promoting hepatic inflammation [85]. Conversely, PXR activation has also been shown to stimulate the NLRP3 inflammasome, leading to caspase-1 activation and subsequent cleavage and maturation of interleukin-1β (IL-1β), thereby enhancing proinflammatory signaling [86,87] (Figure 5). These findings highlight the dual role of PXR in inflammation regulation, where both inhibition and activation of PXR can exacerbate inflammatory responses through distinct pathways. This paradox may stem from the context-dependent nature of PXR activation: low-dose or selective ligands may preferentially induce anti-inflammatory pathways via NF-κB suppression, whereas strong or sustained activation may promote inflammasome priming and lipotoxic stress. This duality is further supported by evidence from other studies. For instance, PXR activation in HepG2 cells upregulates the expression of sterol regulatory element-binding protein 1 (SREBP1), promoting hepatic lipogenesis and lipid accumulation. Conversely, knockdown of PXR in HepG2 cells results in increased expression of aldo-keto reductase 1B10 (AKR1B10), which stabilizes acetyl-CoA carboxylase (ACC), a rate-limiting enzyme in de novo lipogenesis, thereby enhancing fatty acid synthesis and contributing to steatosis [88] (Figure 5). Taken together, these findings underscore the critical importance of maintaining PXR homeostasis in preventing the onset and progression of MASLD. Therapeutic strategies aimed at fine-tuning PXR activity may offer promising avenues for the management of this increasingly prevalent metabolic liver disorder.

### 3.5. Cancer

Over the past several decades, cancer has remained a leading cause of mortality worldwide [89]. The development of human cancers is the result of a series of progressive cellular alterations. Given the high frequency of cellular division and the accumulation of mutagenic events throughout the human lifespan, although the majority of mutations are benign, a subset may escape immune surveillance, leading to uncontrolled proliferation and eventual transformation into malignant cells, culminating in tumor formation [90,91,92]. Hepatocellular carcinoma (HCC), the most prevalent type of primary liver cancer, is currently the second leading cause of cancer-related deaths globally, posing a substantial threat to human health. Therefore, elucidating the mechanisms underlying HCC development is of critical importance [93].

**Figure 6:**
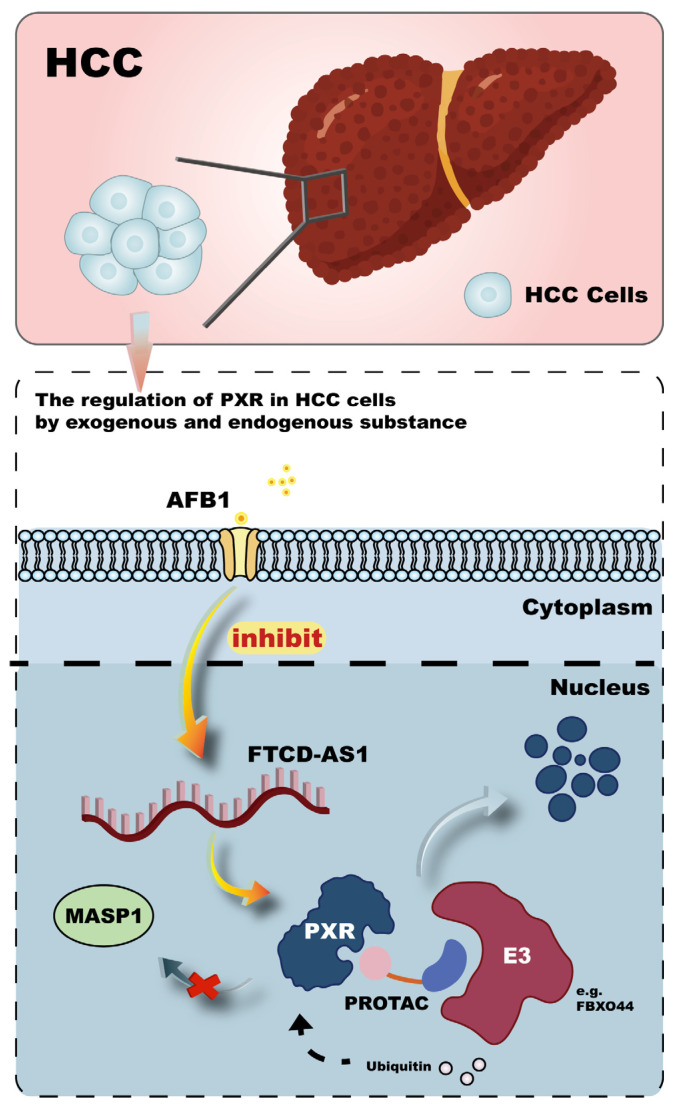
Mechanisms of PXR’s action in hepatocellular carcinoma. The figure indicates that AFB1 (aflatoxin B1) exposure can modulate PXR and suppress the FTCD-AS1 (FTCD antisense RNA 1)-PXR-MASP1 (mannan-binding lectin serine protease 1) axis, thereby potentiating HBV-related hepatic injury and the progression to HCC. To counteract PXR-mediated chemoresistance, the diagram introduces the concept of PROTACs (proteolysis-targeting chimeras), which induce targeted ubiquitination and proteasomal degradation of PXR.

Recent studies have increasingly focused on the pathological regulation of the PXR in malignancies and its association with hepatic injury and enhanced HCC risk. One study established a tree shrew model carrying a hepatitis B virus (HBV) plasmid and exposed to aflatoxin B1 (AFB1), revealing significant inhibition of PXR expression. In vitro, AFB1 exposure in HBV-integrated Hep3B and HepG2.215 cells further modulated PXR and suppressed the FTCD antisense RNA 1 (FTCD-AS1)-PXR-mannan-binding lectin serine protease 1 (MASP1) axis (Figure 6). This study demonstrated a mechanistic link by which AFB1 potentiates HBV-related hepatic injury and progression to HCC [94]. Furthermore, environmental pollutants have also been implicated as key factors contributing to HCC pathogenesis. For instance, perfluoroether sulfonic acid BP2 (PFESA-BP2), a fluorinated ether sulfonic acid possibly found in drinking water, has been shown at low doses to activate signaling pathways such as NOTCH4, HIF, and EGF, thereby increasing the risk of HCC [95]. Diethylnitrosamine (DEN), a classic chemical used to induce liver cancer models, has also been found to suppress Akr1c family members, particularly Akr1c18, in PXR knockout mice, resulting in decreased PGF2α levels and ultimately promoting HCC development [96]. These findings not only provide novel therapeutic targets but also enrich the mechanistic understanding of hepatocarcinogenesis. From a public health perspective, they highlight the necessity of minimizing environmental exposure and enhancing pollutant surveillance to protect human health.

Beyond its role in tumorigenesis, PXR also plays a central role in mediating chemoresistance in cancer therapy. A deeper understanding of PXR-regulated mechanisms in non-HCC malignancies may provide new perspectives for HCC research. Chemoresistance, characterized by diminished cancer cell sensitivity to chemotherapy, involves multiple mechanisms including enhanced drug efflux, DNA repair, and inhibition of apoptosis [97]. In triple-negative breast cancer (TNBC), which lacks targeted therapies, resistance to paclitaxel has been attributed to overexpression of the catalytic core PSEN-1, enhancing γ-secretase activity and promoting nuclear accumulation of Notch intracellular domain (NICD). NICD then interacts with and activates PXR, leading to upregulation of multidrug resistance genes and heightened resistance to paclitaxel [98]. Additionally, TPX2, a microtubule-associated protein regulated by paclitaxel, has been shown to recruit PXR to the PXRE or XREM regions of the CYP3A4 promoter, thereby enhancing its transcription and accelerating paclitaxel metabolism, contributing to drug resistance [99]. To counteract PXR-mediated chemoresistance, a novel class of therapeutics known as proteolysis-targeting chimeras (PROTACs) has emerged. These compounds consist of a PXR ligand, a linker, and an E3 ubiquitin ligase ligand, which collectively induce targeted ubiquitination and proteasomal degradation of PXR, thereby blocking the transcriptional activation of drug-metabolizing enzymes and enhancing the antitumor efficacy of paclitaxel [100,101] (Figure 6). The design of effective PROTACs relies heavily on optimizing linker length and chemical properties. A recent study identified F-box protein 44 (FBXO44) as a novel E3 ligase for PXR [102]. Elucidating the structure-function relationship between FBXO44 and PXR could provide valuable insights for the rational design of more efficient PROTACs aimed at overcoming chemoresistance in cancer therapy.

Tumor metastasis, a major cause of cancer-related mortality, remains a significant clinical challenge. It is defined as the dissemination of cancer cells from the primary site to distant organs [103,104]. Epithelial-mesenchymal transition (EMT) has been identified as a key mechanism facilitating invasion and metastasis in HCC [105]. In human hepatic stellate cell-derived hPXR-LX2 cell lines, activation of hPXR was found to inhibit TGF-β1–induced EMT by suppressing the NF-κB-mediated transcription of POSTN, thereby reducing EMT and limiting metastatic potential [106]. Moreover, PXR may exert anti-metastatic effects through modulation of the tumor microenvironment, further supporting its therapeutic potential in cancer [107]. The seemingly paradoxical effects of PXR activation, which can both promote chemoresistance and inhibit epithelial-mesenchymal transition (EMT) and metastasis, can be elucidated through a multifaceted perspective. Specifically, cellular heterogeneity may be a crucial factor, with distinct cell types exhibiting divergent responses to PXR activation. The current landscape of targeted therapy research for hepatocellular carcinoma (HCC) still harbors significant gaps, presenting a promising avenue for further exploration. Future studies should focus on elucidating the response mechanisms of PXR in human HCC cells to various targeted drug ligands and integrate these findings with other PXR—mediated biological processes, such as EMT. This integrative approach may pave the way for novel therapeutic strategies against cancer.

Targeting key molecular nodes into cancer for precision therapy remains a top priority. Integrated omics approaches combined with large-scale CRISPR-Cas9 screening have yielded comprehensive cancer target atlases [108]. Recent work identified LIM and SH3 protein 2 (LASP2) as a novel transcriptional target of PXR. PXR activation significantly upregulated LASP2 expression in murine liver [109]. LASP2 has been shown to exert context-dependent roles across various cancer types [110,111,112]. In HCC, LASP2 expression is significantly downregulated, and its restoration suppresses the MAPK/ERK pathway, reduces HepG2 cell viability, colony formation, and migratory capacity [113]. These findings suggest that PXR-mediated LASP2 overexpression may represent a novel therapeutic strategy for HCC, thereby providing new insights into the development of diagnostic and therapeutic tools targeting nuclear receptors such as PXR.

In addition to HCC, PXR has also been implicated in chemoresistance and redox regulation in other malignancies, thereby contributing to the broader framework of PXR’s oncological roles. For instance, 5-fluorouracil (5-FU), a key chemotherapeutic agent for colorectal cancer (CAC), frequently induces resistance in clinical settings. A study demonstrated that co-treatment with the traditional Chinese medicine formula Fengliang Changweikang (FLCWK) significantly reduced 5-FU-induced PXR activation, downregulated multidrug resistance gene 1 (MDR1) and P-glycoprotein (P-gp), and inhibited the IL-6/STAT3 signaling pathway, thus sensitizing cancer cells to 5-FU [114]. These findings underscore the therapeutic potential of modulating PXR activity to overcome chemoresistance. However, future studies should focus on identifying and characterizing the bioactive components of combination therapies and constructing detailed PXR-regulated resistance networks. Further evidence indicates that PXR activation accelerates chemoresistance through upregulation of drug-metabolizing enzymes, conjugating enzymes, and efflux transporters, including ATP-binding cassette (ABC) transporters, thereby promoting rapid drug clearance in malignancies such as osteosarcoma [115,116]. These insights highlight the complexity of PXR-mediated chemoresistance and reinforce the necessity of multi-faceted investigations to refine therapeutic strategies targeting PXR.

## 4. The Therapeutic Potential of PXR as a Drug Target

### 4.1. PXR Agonists and Antagonists

PXR agonists function by binding to and activating the receptor, while antagonists suppress receptor activity. These modulators influence the expression of downstream target genes such as CYP3A4 and MDR1, thereby impacting drug metabolism and transport. The identification and application of selective and potent PXR agonists or antagonists is essential for precisely elucidating the physiological and pathological roles of PXR in humans, ultimately enabling the development of novel therapeutic strategies. We have summarized representative PXR modulators reported in clinical or preclinical studies to guide future investigations. Based on a comprehensive literature search, we listed five major PXR agonists currently used in research [21,117,118,119,120], as well as four reported antagonists [121,122,123,124] (Table 1). Our findings indicate that only a few PXR agonists have advanced into clinical use, with most still at the preclinical stage, primarily aimed at understanding PXR’s mechanistic roles. This highlights a significant translational barrier that must be addressed. For instance, rifampicin, a well-characterized agonist, exhibits species- and dose-dependent dual effects—activating PXR at lower doses, yet inhibiting it under certain conditions—posing potential risks in clinical application [21,125]. This complexity underscores the importance of rigorous preclinical evaluation of known modulators before their therapeutic use.

Beyond the listed compounds (Table 1), various other synthetic and natural molecules have been investigated for their ability to modulate PXR activity. Natural products, in particular, represent a promising but underexplored category of PXR modulators. Hyperforin, a well-known PXR agonist, has established a benchmark for natural product-based research in this field [118]. A study identified eight non-aromatic acylphloroglucinol–terpenoid adducts isolated from *Hypericum perforatum*, four of which demonstrated stronger activation of PXR compared to rifampicin. Molecular docking analysis suggested that these compounds interact with key residues within the PXR ligand-binding domain (LBD) via hydrogen bonding [126]. Similarly, cannabidiol (CBD) has been reported as a species-selective PXR agonist, with greater activation capacity for human PXR than for rodent PXR, contrasting with pregnenolone 16α-carbonitrile (PCN). Resveratrol (RES), another natural product, was employed in comparative studies as a PXR antagonist [69,117]. Additional research identified furanodienone (FDN), a major sesquiterpenoid in Zingiber species, as a potential PXR agonist that binds to and alters the structure of the PXR-LBD, modulating downstream gene expression [100,127]. Paclitaxel, a chemotherapeutic agent, has also been implicated in PXR activation. These findings highlight the therapeutic promise of natural compounds in modulating PXR activity; however, their simultaneous use warrants careful investigation due to the risk of synergistic overactivation or inhibition that could disrupt PXR-mediated pathways. Considering the pivotal role of natural products in unleashing the therapeutic potential of PXR as a drug target, particularly in the context of metabolic diseases, traditional Chinese medicines (TCMs) may hold unique promise for future drug discovery endeavors. Boasting a long-standing history of clinical application and a wealth of pharmacological insights, TCMs are gaining increasing recognition for their potential contributions to modern medicine. For example, specific Chinese herbal formulations, such as Fengliang Changweikang, have demonstrated the ability to reverse chemoresistance through PXR modulation, thereby underscoring the potential of TCMs in addressing metabolic disorders and associated drug resistance challenges [114]. The convergence of multi-omics and network pharmacology approaches could further augment the screening and identification of selective PXR modulators derived from traditional herbal compounds, thereby offering novel perspectives for the development of innovative therapeutic strategies targeting metabolic diseases.

In parallel, synthetic small molecules have shown considerable promise in PXR modulation. MI-883, for example, is a rationally designed dual-function compound that acts as a PXR antagonist and a constitutive CAR agonist, offering the potential to coordinately regulate overlapping gene networks controlled by these two nuclear receptors [74]. IPA, an indole derivative produced by gut microbiota via tryptophan metabolism, is a well-characterized endogenous PXR agonist [128]. Building on this scaffold, a novel microbial metabolite mimic, FKK6, was designed to serve as a PXR agonist with anti-inflammatory and tissue-protective effects. Notably, FKK6 demonstrated high intestinal absorption but rapid hepatic clearance, suggesting tissue-specific pharmacokinetics [129,130]. Another synthetic PXR agonist, tributyl citrate (TBC), was similarly reported to exhibit intestinal tissue specificity [51] (Table 1). These data collectively indicate the feasibility of rational molecular design to achieve controlled modulation of PXR activity, while also emphasizing the importance of evaluating species and tissue selectivity in drug development.

As technology and screening methodologies evolve, the identification of novel PXR modulators remains a critical focus. High-throughput screening (HTS) platforms have been employed to identify candidate PXR antagonists, followed by validation through cell-based assays and molecular docking to isolate compounds with true inhibitory efficacy [131]. Co-crystal structure analysis of PXR-ligand complexes has emerged as a powerful tool to reveal the molecular basis of ligand binding and receptor modulation. Recent structural studies have shown that several azole-based antagonists bind to a hydrophobic pocket near the AF-2 allosteric site on the PXR surface, distinct from the binding site of typical agonists [132]. Co-crystal structures help elucidate the conformational changes induced by antagonists and provide insights into the structural determinants of selectivity. Such findings form the theoretical basis for the rational design of next-generation PXR modulators aimed at mitigating undesirable drug–drug interactions by controlling PXR-mediated gene regulation [25,133]. Collectively, these advances emphasize that the successful development of PXR agonists and antagonists must integrate structural biology, computational modeling, and functional validation. Attention to dynamic allosteric effects and binding site plasticity will be essential for the precise design and screening of therapeutically relevant PXR modulators.

### 4.2. Challenges in Drug Development

Previous studies have clearly established the pivotal role of the pregnane X receptor (PXR) in drug metabolism. PXR modulates the expression of various drug-metabolizing enzymes (DMEs), including both phase I and phase II enzymes, as well as drug transporters [134]. Among phase I enzymes, cytochrome P450s (CYPs) are the most critical and well-characterized targets downstream of PXR. Notably, members of the CYP1, CYP2, and CYP3 families—such as CYP3A4—are key mediators of xenobiotic metabolism, catalyzing oxidation, reduction, and hydrolysis reactions that facilitate drug clearance [135,136,137]. In addition, phase I enzymes work in concert with phase II enzymes, such as UDP-glucuronosyltransferase 1A1 (UGT1A1), and transport proteins like P-glycoprotein (P-gp), both of which are also under the regulatory control of PXR [138,139]. Dysregulation of these proteins may lead to significant drug–drug interactions (DDIs), ultimately contributing to adverse events such as drug-induced liver injury (DILI).

DILI is an adverse hepatic reaction triggered by potentially hepatotoxic drugs [140]. PXR plays a crucial role in this process by regulating the transcription of numerous CYP isoforms, including CYP3A4, CYP3A7, CYP2B6, CYP2C8, and CYP2C9, all of which are involved in the metabolism of endogenous and exogenous compounds [141]. Furthermore, PXR also modulates the expression of UGT1A1 and several drug transporters such as multidrug resistance protein 1 (MDR1) and ATP-binding cassette subfamily C member 2 (MRP2/ABCC2), which contribute to drug detoxification and maintenance of tissue homeostasis [142]. Among these, CYP3A4 is particularly noteworthy for its ability to metabolize approximately 60% of all clinically used drugs. PXR activation upregulates CYP3A4 expression, thereby increasing the risk of clinically significant DDIs [21]. Acetaminophen (APAP) is a widely used over-the-counter antipyretic and analgesic agent that, in overdose scenarios, is a major cause of DILI, leading to severe hepatic dysfunction. Recent studies have shown that suppression of PXR activation can reduce the expression of CYP1A2, CYP3A11, and CYP3A4, consequently decreasing the formation of hepatotoxic APAP metabolites. In addition, inhibition of PXR activation attenuates endoplasmic reticulum stress (ERS) by downregulating the PERK–eIF2α–ATF4 signaling axis and suppressing the expression of pro-apoptotic factors such as Caspase-3/7, thereby mitigating ERS-induced hepatocyte apoptosis and liver injury [143,144]. Interestingly, exposure to APAP has also been shown to induce S-nitrosylation of PXR (SNO-PXR) in murine liver, and elevated levels of SNO-PXR were associated with enhanced hepatic protection, revealing a novel post-translational regulatory mechanism for ameliorating DILI [145]. Given the unpredictable nature of DILI, the development of predictive tools for assessing drug toxicity is of critical importance. A recent study has introduced a transgenic zebrafish embryo model, tg (cyp3a65:GFP), to screen metabolism-disrupting chemicals (MDCs) and evaluate their toxicological risk [146]. This model provides a novel in vivo platform for investigating drug-induced hepatotoxicity. It is conceivable that the construction of similar experimental models, centered on elucidating PXR-mediated mechanisms, will significantly enhance the predictive accuracy of DILI risk assessment. However, this endeavor necessitates further refinement of the PXR regulatory network.

Notably, DILI represents only one facet of the broader challenges encountered in PXR-targeted drug development. Structural complexity and ligand-binding promiscuity of PXR pose significant hurdles in the design of specific and efficacious agonists or antagonists. The development of PXR antagonists is particularly underexplored, with few candidates achieving clinical applicability [147]. Additionally, the tissue-specific and species-specific nature of PXR function limits the generalizability of findings and complicates both experimental design and translational research. These challenges underscore the need for precise experimental modeling, careful selection of preclinical systems, and innovative strategies to improve the success rate of drug discovery targeting PXR.

### 4.3. Novel Therapeutic Strategies and Multi-Omics Approaches

Given the pivotal role of PXR in maintaining physiological homeostasis and regulating drug metabolism, the development of innovative research methodologies is essential. The CRISPR/Cas9 genome editing system has enabled the generation of PXR-knockout models, facilitating the exploration of novel PXR-associated signaling pathways and the refinement of its regulatory network [148]. In addition, recent advances in genome editing, including base editors and prime editors, have overcome the limitations associated with double-strand breaks (DSBs) induced by conventional CRISPR/Cas9 techniques, thereby enhancing the safety and precision of site-specific genetic modifications [149]. To overcome species-specific differences in PXR function and reduce interspecies variability in mechanistic studies, hPXR mouse models have been developed for in vivo experiments. These models allow for more accurate predictions of drug–drug interaction (DDI) risks in patients undergoing polypharmacy [150]. However, such models remain imperfect, as they do not fully address species-specific variations in upstream regulatory mechanisms or downstream gene expression profiles of PXR. Therefore, further refinement of humanized models or the development of alternative strategies is warranted.

Beyond genetic approaches, nanotechnology-based delivery systems have emerged as promising therapeutic platforms. For example, a nanoparticle system composed of ketoconazole (KCZ) conjugated with endothelin-2 (NPs/KCZ) has been engineered to effectively inhibit PXR overactivation and enable targeted delivery for the treatment of drug-resistant epilepsy (DRE) [151]. Similarly, the combination of paclitaxel (PTX) with the PXR antagonist SPA70 has been demonstrated to overcome chemoresistance in non-small cell lung cancer (NSCLC) [152]. These studies underscore the therapeutic potential of integrating nanomedicine and combinational therapy, particularly in PXR-targeted treatment paradigms. In future research, the encapsulation of PXR antagonists such as SPA70 within lipid-based nanoparticles or liposomes could enhance tumor targeting while minimizing off-target effects.

A comprehensive understanding of the PXR regulatory network is fundamental to the design of novel therapeutic strategies. Multi-omics technologies—including genomics, transcriptomics, proteomics, and metabolomics—are essential for decoding the complexity of PXR-mediated signaling. For instance, pharmacogenomics studies using genome-wide association studies (GWAS) have identified genomic loci associated with PXR activity and delineated the precise binding sites of rifampicin, a well-known PXR agonist [153]. Additionally, chromatin immunoprecipitation followed by high-throughput sequencing (ChIP-Seq) has enabled genome-wide mapping of PXR-binding sites in mouse liver, providing a detailed atlas of its genomic interactions [154]. Transcriptomic analyses, particularly RNA-Seq, have been employed to characterize downstream gene expression profiles following PXR activation or inhibition, thereby uncovering novel signaling pathways or refining established mechanisms. For example, previous studies using RNA-Seq have elucidated the downstream targets of PXR and CAR in hepatic tissues [155]. Post-translational modifications (PTMs), studied through proteomic approaches, represent another key regulatory dimension of PXR. Modifications such as phosphorylation, ubiquitination, and acetylation significantly influence PXR’s transcriptional activity, stability, subcellular localization, and interactions with ligands or heterodimeric partners like RXR [156]. Taken together, multi-omics approaches provide complementary insights and enable the systematic reconstruction of the PXR regulatory network. These integrative strategies pave the way for a deeper understanding of PXR biology and inform the development of personalized therapeutic interventions, thereby overcoming the limitations of single-omics analyses in decoding complex biological systems.

## 5. Future Research Directions

### 5.1. Interactions Between PXR and the Gut Microbiota

Recent studies have revealed that tryptophan (TRP) can be metabolized via three major pathways: the serotonin (5-HT) pathway, the kynurenine pathway (KP), and the gut microbiota-dependent pathway, with the latter playing a critical role [157]. Notably, numerous investigations have demonstrated that intestinal microbiota can convert TRP into IPA, a potent endogenous agonist of PXR [128]. Theoretically, circulating IPA levels may serve as a biomarker to predict the risk of developing MASLD, T2DM, and cardiovascular diseases [157]. For instance, in cardiovascular diseases-related studies, elevated plasma IPA levels have been shown to activate PXR in platelets, subsequently inhibiting downstream signaling pathways and suppressing platelet activation, thereby effectively preventing thrombosis [66]. Furthermore, PXR gene knockout in murine fibroblasts exacerbated fibrosis after dextran sulfate sodium (DSS)-induced colitis, whereas IPA administration attenuated fibrotic progression. Additionally, PXR expression was markedly reduced in intestinal biopsy specimens from patients with Crohn’s disease (CD) and ulcerative colitis (UC) [158]. These findings underscore the importance of maintaining a balanced composition and abundance of gut microbiota to ensure adequate IPA production, which is essential for endogenous PXR activation and the modulation of disease-related downstream pathways. A recent study identified a novel indole-analogous microbial metabolite, FKK6, as a PXR agonist. Experimental validation indicated that FKK6 has no mutagenic potential and exhibits gut specificity, offering a new tool for probing microbiota–PXR interactions [129,130].

In parallel, *Clostridium sporogenes* (*C. spor.*) was shown to be a key bacterium responsible for IPA production, which was significantly reduced in ovariectomized mice. A silk protein-based hydrogel encapsulating *C. spor.* was developed to enhance its intestinal colonization and IPA biosynthesis, serving as an alternative to conventional microbial transplantation and promoting PXR–p65 interactions to maintain skeletal homeostasis [159]. These findings highlight an emerging research avenue: how to effectively modulate gut microbial composition to exert beneficial PXR-mediated effects. A recent innovation combined probiotics, prebiotics, and nanocarriers to develop a “nano-synbiotic” system, capable of precise targeting and modulation of microbial structure [160]. While promising, further investigation is needed to delineate the specific regulatory mechanisms of diverse microbial taxa and their bioactive metabolites, thereby constructing a comprehensive microbiota–PXR interaction network.

In addition to TRP metabolism, gut microbiota can regulate PXR activity by modulating bile acid (BA) metabolism. Reverse metabolomic profiling revealed that certain gut microbes, such as *Bifidobacterium* and *Clostridium* species, produce amino acid-conjugated BAs that are elevated in inflammatory bowel disease (IBD) [161]. Moreover, bacterial bile salt hydrolase (BSH) not only catalyzes bile acid deconjugation but also acts as an amine N-acyltransferase to generate bacterial bile acid amides (BBAAs), which have been shown to directly activate PXR [162]. These results highlight the therapeutic potential of targeting microbial enzymes and their metabolites to restore PXR signaling homeostasis in inflammation-associated diseases. Future studies should explore the structural diversity of BAs produced by different microbial taxa and their tissue-specific interactions with host receptors.

However, PXR functions as a double-edged sword. For example, Atractylodes macrocephala Koidz polysaccharide (PAMK) ameliorated DSS-induced colitis by modulating gut microbiota, enhancing TRP metabolism, and activating PXR to suppress intestinal inflammation [163]. Conversely, antibiotic-mediated depletion of the gut microbiota significantly mitigated PXR agonist PCN-induced hepatomegaly and downregulated PXR, YAP, and their downstream proteins, suggesting that PXR activation may contribute to hepatic enlargement under dysbiotic conditions [164]. These observations emphasize the necessity of maintaining the balance between PXR homeostasis and microbial architecture to avoid adverse outcomes in various tissues.

### 5.2. Personalized Medicine

The NR1I2 gene encoding PXR exhibits extensive single nucleotide polymorphisms (SNPs), with over 1800 variants recorded in the dbSNP database as of 2017. These polymorphisms may significantly influence the expression of downstream drug-metabolizing enzymes, necessitating personalized therapeutic strategies [165]. In a recent clinical study, the metabolic clearance of midazolam in mechanically ventilated intensive care unit (ICU) patients was found to be significantly associated with NR1I2 rs2461817 polymorphism, indicating the need for individualized midazolam dosing [166]. Another variant, NR1I2 rs13059232, has been identified as a potential biomarker in patients receiving clopidogrel and antiplatelet therapy for acute ischemic stroke [167]. Deciphering NR1I2 polymorphisms is pivotal to advancing N-of-1 trial designs and developing patient-centric treatments tailored to dynamic molecular landscapes [168].

Moreover, patients with chronic microenvironmental disturbances may exhibit altered PXR expression. In uremic patients, the accumulation of protein-bound uremic toxins (PBUTs) disrupts the systemic environment and suppresses CYP3A4 expression via the PXR/NF-κB pathway, impairing the metabolism of atorvastatin [169]. Similarly, elevated mRNA levels of drug-metabolizing enzymes and multidrug resistance proteins in hypertensive nephropathy (HN) have been attributed to aberrant PXR activation, potentially compromising the efficacy of combination pharmacotherapy [170].

## 6. Conclusions

PXR shares structural homology with other nuclear receptors, but its ligand-binding domain (LBD) is uniquely composed of a helical sandwich structure with a distinctive insertion loop and a tryptophan zipper motif. These features confer PXR with a flexible and promiscuous ligand-binding pocket, enabling it to interact with a wide range of endogenous and exogenous compounds. Consequently, PXR is highly susceptible to activation or inhibition by xenobiotics such as drugs and environmental pollutants, as well as endogenous metabolites like bile acids during physiological perturbations. The ligand-dependent activation and suppression of PXR, as well as post-translational modifications (e.g., phosphorylation), modulate the transcription of its downstream targets, primarily drug-metabolizing enzymes such as cytochrome P450s (e.g., CYP3A4) and transporters like P-glycoprotein (P-gp) encoded by MDR1. The dysregulation of these pathways can lead to insulin resistance, aberrant gluconeogenesis, and adverse drug–drug interactions, contributing to chronic diseases such as T2DM, obesity, atherosclerosis, and MASLD, and cancer. Therefore, maintaining PXR homeostasis is critical for human health.

Integrating gene-editing technologies and multi-omics approaches will be crucial for elucidating PXR-regulated networks and facilitating the rational design of clinical PXR agonists and antagonists. This will also aid in predicting drug–drug interactions and developing novel therapeutic strategies. The gut microbiome is emerging as a key modulator of PXR, with microbial metabolites such as IPA serving as vital endogenous ligands, thereby strengthening the rationale for microbiota-targeted therapies. The interindividual variation in gut microbial composition and NR1I2 gene polymorphisms further underscores the importance of precision medicine. In patients with chronic diseases, disrupted homeostasis may alter PXR-regulated gene expression, reinforcing the potential of N-of-1 trials.

Future directions for PXR research include structural biology studies to understand ligand-induced conformational changes and reduce off-target effects; the species- and tissue-specific characterization of PXR to refine experimental models; the exploration of the epigenetic landscape and NR1I2 polymorphisms in disease contexts; and the development of organoid and organ-on-chip systems (e.g., gut organoids) to dissect microenvironmental influences on PXR. The application of single-cell sequencing, spatial transcriptomics, and artificial intelligence-based predictive modeling will further propel breakthroughs in the PXR research landscape. We envision that interdisciplinary and multi-sectoral collaborations will accelerate advances in this field, ultimately contributing to the improvement of global health outcomes.

## Figures and Tables

**Table 1:** Overview of representative PXR modulators, their pharmacological characteristics, and potential clinical applications.

Compound	Class	Role	Research Stage	Potential Applications	Reference
Rifampicin	Antibiotic	Agonist	Clinical	Anti-tuberculosis therapy; regulation of drug metabolism	[21]
Pregnenolone-16α-carbonitrile (PCN)	Synthetic steroid	Agonist	Preclinical	Species-specific PXR activator commonly used in rodent models	[117]
Hyperforin	Natural product	Agonist	Clinical	Treatment of depression; modulation of drug metabolism	[118]
SR12813	Synthetic small molecule	Agonist	Preclinical	Activation of PXR; regulation of cholesterol metabolism	[119]
Highly Chlorinated Polychlorinated Biphenyls (PCBs)	Industrial pollutant	Agonist	Preclinical	Activation of PXR-mediated xenobiotic responses	[120]
Ketoconazole	Antifungal drug	Antagonist	Clinical (withdrawn due to hepatotoxicity)	Inhibits the PXR–CYP3A4 axis; antifungal agent	[121]
Berberine	Natural alkaloid	Antagonist	Preclinical	Sensitizes colorectal cancer to chemotherapy; reverses P-gp/CYP3A4-mediated resistance	[122]
SPA70	Synthetic small molecule	Antagonist	Preclinical	Competitively binds to the PXR ligand-binding domain, preventing agonist-induced activation	[123]
SPB03255	Synthetic small molecule	Antagonist	Preclinical	Inhibits PXR activation	[124]
The newly identified components from Hypericum japonicum	Natural products	Agonist	Preclinical	Potential therapeutic agents for cholestasis via PXR activation	[126]
Cannabidiol (CBD)	Natural products	Agonist	Preclinical	Selective PXR agonist; exhibits high activity toward hPXR	[69]
Resveratrol (RES)	Natural products	Antagonist	Preclinical	Suppresses PXR-mediated downstream gene expression; binds to non-canonical sites on PXR	[69]
Furanodienone (FDN)	Natural products	Agonist	Preclinical	Activates PXR via unique interaction with LBD, inducing conformational changes	[127]
Paclitaxel	Natural products	Agonist	Preclinical	Anticancer agent; induces PXR activation and contributes to chemoresistance	[100]
MI-883	Synthetic small molecule	Antagonist	Preclinical	Modulates downstream signaling pathways via dual regulation of PXR and CAR	[74]
IPA	Indole derivative	Agonist	Preclinical	Microbiota-derived PXR agonist; regulates intestinal barrier function	[128]
Felix Kopp Kortagere 6 (FKK6)	Synthetic small molecule	Agonist	Preclinical	Selectively activates intestinal PXR; exhibits anti-inflammatory effects	[129]

## Abbreviations

The following abbreviations are used in this manuscript:

PXR	pregnane X receptor
T2DM	type 2 diabetes mellitus
AS	atherosclerosis
MASLD	metabolic dysfunction-associated steatotic liver disease
NRs	nuclear receptors
DBD	DNA-binding domain
LBD	ligand-binding domain
ZF	zinc finger
hPXR-LBD	human PXR LBD
Trp-Zip	tryptophan zipper
RXR	retinoid X receptor
CYP	cytochrome P450
PCN	pregnenolone 16α-carbonitrile
AF	activation function
hRXRα	human RXRα
PXRE	ER6-type PXR response element
P-gp	P-glycoprotein
MRP2	multidrug resistance-associated protein 2
ABC	ATP-binding cassette
SRC1	steroid receptor coactivator-1
MDR1	multidrug resistance protein 1
CESs	carboxylesterases
JNK	c-Jun N-terminal kinase
PXR-KO	PXR-knockout
HFD	high-fat diet
CAR	constitutive androstane receptor
FOXO1	forkhead box protein O1
G6P	glucose-6-phosphatase
PEPCK1	phosphoenolpyruvate carboxykinase 1
HNF4α	hepatocyte nuclear factor 4 alpha
GLUT2	glucose transporter 2
PCBs	polychlorinated biphenyls
NDL-PCBs	non-dioxin-like PCBs
PBDEs	polybrominated diphenyl ethers
IPA	indole-3-propionic acid
B3galt5	β-1,3-galactosyltransferase 5
LPS	lipopolysaccharide
NF-κB	nuclear factor-kappa B
AP-1	activator protein-1
LDL-C	low-density lipoprotein cholesterol
DIT	diffuse intimal thickening
SFKs	Src family kinases
IF	Intermittent fasting
HSP90	heat shock protein 90
SREBP2	sterol regulatory element-binding protein 2
NAFLD	non-alcoholic fatty liver disease
MPHs	primary human hepatocytes
SLC27A4	solute carrier family 27 member 4
PPARs	peroxisome proliferator-activated receptors
FATPs	fatty acid transport proteins
LCFAs	long-chain fatty acids
MASH	Metabolic dysfunction-associated steatohepatitis
IL-6	interleukin-6
TNF-α	tumor necrosis factor-α
IL-1β	interleukin-1β
SREBP1	sterol regulatory element-binding protein 1
AKR1B10	aldo-keto reductase 1B10
ACC	acetyl-CoA carboxylase
HCC	Hepatocellular carcinoma
HBV	hepatitis B virus
AFB1	aflatoxin B1
FTCD-AS1	FTCD antisense RNA 1
MASP1	mannan-binding lectin serine protease 1
PFESA-BP2	perfluoroether sulfonic acid BP2
TNBC	triple-negative breast cancer
NICD	Notch intracellular domain
PROTACs	proteolysis-targeting chimeras
FBXO44	F-box protein 44
EMT	Epithelial-mesenchymal transition
LASP2	LIM and SH3 protein 2
5-FU	5-fluorouracil
CAC	colorectal cancer
FLCWK	Fengliang Changweikang
TBC	tributyl citrate
HTS	High-throughput screening
FKK6	Felix Kopp Kortagere 6
DMEs	drug-metabolizing enzymes
UGT1A1	UDP-glucuronosyltransferase 1A1
DDIs	drug–drug interactions
DILI	drug-induced liver injury
ERS	endoplasmic reticulum stress
MDCs	metabolism-disrupting chemicals
DSBs	double-strand breaks
DRE	drug-resistant epilepsy
NSCLC	non-small cell lung cancer
GWAS	genome-wide association studies
PTMs	Post-translational modifications
DSS	dextran sulfate sodium
UC	ulcerative colitis
BA	bile acid
IBD	inflammatory bowel disease
BSH	bacterial bile salt hydrolase
ZBBAAs	bacterial bile acid amides
SNPs	single nucleotide polymorphisms
HN	hypertensive nephropathy
